# Impact of nutrient excess and endothelial nitric oxide synthase on the plasma metabolite profile in mice

**DOI:** 10.3389/fphys.2014.00453

**Published:** 2014-11-25

**Authors:** Brian E. Sansbury, Aruni Bhatnagar, Bradford G. Hill

**Affiliations:** ^1^Division of Cardiology, Department of Medicine, Institute of Molecular Cardiology, University of LouisvilleLouisville, KY, USA; ^2^Department of Medicine, Diabetes and Obesity Center, University of LouisvilleLouisville, KY, USA; ^3^Department of Physiology and Biophysics, University of LouisvilleLouisville, KY, USA; ^4^Department of Biochemistry and Molecular Biology, University of LouisvilleLouisville, KY, USA

**Keywords:** metabolomics, diabetes, obesity, nitric oxide, insulin resistance, metabolism

## Abstract

An increase in calorie consumption is associated with the recent rise in obesity prevalence. However, our current understanding of the effects of nutrient excess on major metabolic pathways appears insufficient to develop safe and effective metabolic interventions to prevent obesity. Hence, we sought to identify systemic metabolic changes caused by nutrient excess and to determine how endothelial nitric oxide synthase (eNOS)—which has anti-obesogenic properties—affects systemic metabolism by measuring plasma metabolites. Wild-type (WT) and eNOS transgenic (eNOS-TG) mice were placed on low fat or high fat diets for 6 weeks, and plasma metabolites were measured using an unbiased metabolomic approach. High fat feeding in WT mice led to significant increases in fat mass, which was associated with significantly lower plasma levels of 1,5-anhydroglucitol, lysophospholipids, 3-dehydrocarnitine, and bile acids, as well as branched chain amino acids (BCAAs) and their metabolites. Plasma levels of several lipids including sphingomyelins, stearoylcarnitine, dihomo-linoleate and metabolites associated with oxidative stress were increased by high fat diet. In comparison with low fat-fed WT mice, eNOS-TG mice showed lower levels of several free fatty acids, but in contrast, the levels of bile acids, amino acids, and BCAA catabolites were increased. When placed on a high fat diet, eNOS overexpressing mice showed remarkably higher levels of plasma bile acids and elevated levels of plasma BCAAs and their catabolites compared with WT mice. Treatment with GW4064, an inhibitor of bile acid synthesis, decreased plasma bile acid levels but was not sufficient to reverse the anti-obesogenic effects of eNOS overexpression. These findings reveal unique metabolic changes in response to high fat diet and eNOS overexpression and suggest that the anti-obesity effects of eNOS are likely independent of changes in the bile acid pool.

## Introduction

The relatively recent increase in obesity prevalence is associated with a heightened risk for developing type 2 diabetes (Ervin, 2009; Roger et al., 2012) as well as a risk of developing chronic diseases such as cardiovascular disease and cancer (Calle et al., 1999). In principle, obesity is related to a state of energy imbalance, which is impacted by both nutrient overconsumption or insufficient energy expenditure (Tseng et al., 2010). Excessive caloric intake may be responsible, at least partially, for the high prevalence of obesity in industrialized societies. In the US, the average consumption of calories, derived mostly from high fat-containing foods, has increased by >200 kcal/d per person over the past few decades (Hill and Peters, 1998; Nielsen and Popkin, 2003; Briefel and Johnson, 2004; Kant and Graubard, 2004). The metabolic changes occurring under these conditions are responsible for or permissive in the development of pre-diabetic and diabetic states. Hence, an understanding of how nutrient excess affects systemic and organ-specific metabolism could lead to the identification of new therapeutic targets to prevent obesity and mitigate risk of developing a range of chronic diseases.

Systemic and organ-specific metabolism are regulated by a host of autocrine and paracrine factors such as insulin, adipokines, and nitric oxide (NO). While peptide hormones have been extensively studied, the role of NO in regulating metabolism is less well known. Nevertheless, several studies show that NO bioavailability is decreased in animal models of diabetes and obesity (Bender et al., 2007; Kim et al., 2008) as well as in humans with metabolic disease (Higashi et al., 2001; Gruber et al., 2008). Mice lacking endothelial NO synthase (eNOS), a key source of NO *in vivo*, are insulin resistant, have impaired fatty acid oxidation and display exaggerated high fat diet (HFD)-induced weight gain (Shankar et al., 2000; Duplain et al., 2001; Cook et al., 2003), and, the abundance of eNOS is remarkably diminished in obesity and diabetes (Valerio et al., 2006; Perez-Matute et al., 2009; Georgescu et al., 2011; Kraus et al., 2012; Sansbury et al., 2012). In addition, conditions of nutrient excess lead to uncoupling of eNOS (Cai et al., 2005; Yamamoto et al., 2010; Forstermann and Li, 2011; Kietadisorn et al., 2012; Abudukadier et al., 2013) which increases the production of superoxide and decreases NO bioavailability. These observations suggest that conditions of obesity and diabetes significantly affect NO production and bioavailability, which in turn could attenuate the regulation of metabolism by NO. In support of this view, we have recently reported that genetic overexpression of eNOS (to enhance NO production) in mice prevents diet-induced obesity, increases metabolic activity, and promotes a brown-like adipocyte phenotype in white adipose tissue (Sansbury et al., 2012). Similarly, constitutive activation of eNOS, by knocking in a phosphomimetic point mutation at S1176 of the enzyme, promotes resistance to diet-induced weight gain (Kashiwagi et al., 2013). Hence, both gain- and loss-of-function studies suggest eNOS-derived NO plays a critical role in regulating systemic metabolism.

Although it is evident that NO can markedly affect systemic metabolism, the specific metabolic pathways regulated by NO remain unclear. In cell culture systems, NO has been shown to regulate metabolic pathways such as the hexose monophosphate shunt (Clancy et al., 1997) and glycolysis (Almeida et al., 2004). Nevertheless, whether NO can exert similar metabolic control *in vivo* has not been investigated and specific metabolites affected by NO have not been identified. Moreover, even though NO can promote mitochondrial biogenesis (Nisoli et al., 2003, 2004; Kelly and Scarpulla, 2004), it can paradoxically inhibit mitochondrial respiration acutely by binding to cytochrome oxidase (Shiva et al., 2005; Cooper and Giulivi, 2007). How such diverse actions of NO integrate to regulate systemic metabolism remain to be elucidated. In this study, we used an unbiased metabolomic approach to examine changes in plasma metabolite profiles of wild type (WT) and eNOS transgenic (eNOS-TG) mice fed low fat or high fat diets and determined whether changes in specific metabolic pathways contribute to the lean phenotype of eNOS-TG mice.

## Methods

### Animal studies

All procedures were approved by the University of Louisville Institutional Animal Care and Use Committee. C57BL/6J (WT) and eNOS transgenic (eNOS-TG) mice on the same genetic background were used for all experiments. The eNOS-TG mice express bovine eNOS under the control of the preproendothelin-1 promoter (Ohashi et al., 1998; Sansbury et al., 2012). At 8 weeks of age, male mice were placed on a low (10%) fat diet (LFD; Research Diets, Inc., #D12450B), a high (60%) fat diet (HFD; Research Diets Inc., #D12492) or a custom-formulated HFD containing GW4064, for 6 weeks. The custom GW4064 diet was produced by Research Diets Inc. and was formulated by adding GW4064 (Sigma, #G5172) to the HFD (#D12492) at a concentration of 180 mg of compound/kg of diet, as described previously (Watanabe et al., 2011). Water and food were provided *ad libitum*. Body weights were recorded weekly. At the end of the feeding protocol, body composition analysis and glucose and insulin tolerance tests were performed as described previously (Sansbury et al., 2012).

### Body composition

Body composition was measured by dual-energy X-ray absorptiometry (Dexascan; PIXImus2; Lunar, Madison, WI) as described previously (Sansbury et al., 2012; Cummins et al., 2014).

### Metabolomic analysis of plasma

Whole blood was collected by cardiac ventricular puncture following a 16 h fast. EDTA was added to whole blood samples to prevent coagulation, and plasma was separated from erythrocytes by centrifugation. Samples were stored frozen (−80°C) until metabolite extraction. After extraction in methanol, relative metabolite abundance was measured by GC/MS or LC/MS/MS by Metabolon, Inc. (Durham, NC) as described previously (Cummins et al., 2014; Sansbury et al., 2014). Original scale data (raw area counts) were then analyzed using Metaboanalyst 2.0 software (Xia and Wishart, 2011). Metabolites with missing values were imputed by replacing missing values with half of the minimum positive value in the original data. Metabolites with greater than 50% of the values missing were omitted from the analysis. After a generalized logarithm transformation, the data were autoscaled, i.e., mean-centered and divided by the standard deviation of each variable. After this transformation, the distribution of the intensity values approximated a Gaussian distribution. The transformed values were used for univariate (e.g., volcano plots), multivariate (e.g., PCA), and cluster (heatmap and dendogram) analyses. Z-scores were calculated from raw area count spectral data from those metabolites that attained significance in volcano plot analysis, using the Metaboanalyst 2.0 software (http://www.metaboanalyst.ca/) (Xia and Wishart, 2011).

### Plasma bile acid measurements

Total bile acids were measured using a liquid stable enzymatic colorimetric assay (Randox Laboratories, #BI3863) and analyzed by a Cobas Mira Plus 5600 Autoanalyzer (Roche, Indianapolis, IN).

### Statistical analyses

Data are presented as mean ± s.e.m. Multiple groups were compared using One-Way ANOVA, followed by Bonferroni post-tests. Unpaired Student's *t*-test was used for direct comparisons. For statistical analyses the Metaboanalyst and/or GraphPad 5.0 software was used. A *p* ≤ 0.05 was considered significant.

## Results

### Effects of HFD and eNOS overexpression on obesity

**Figure 1:**
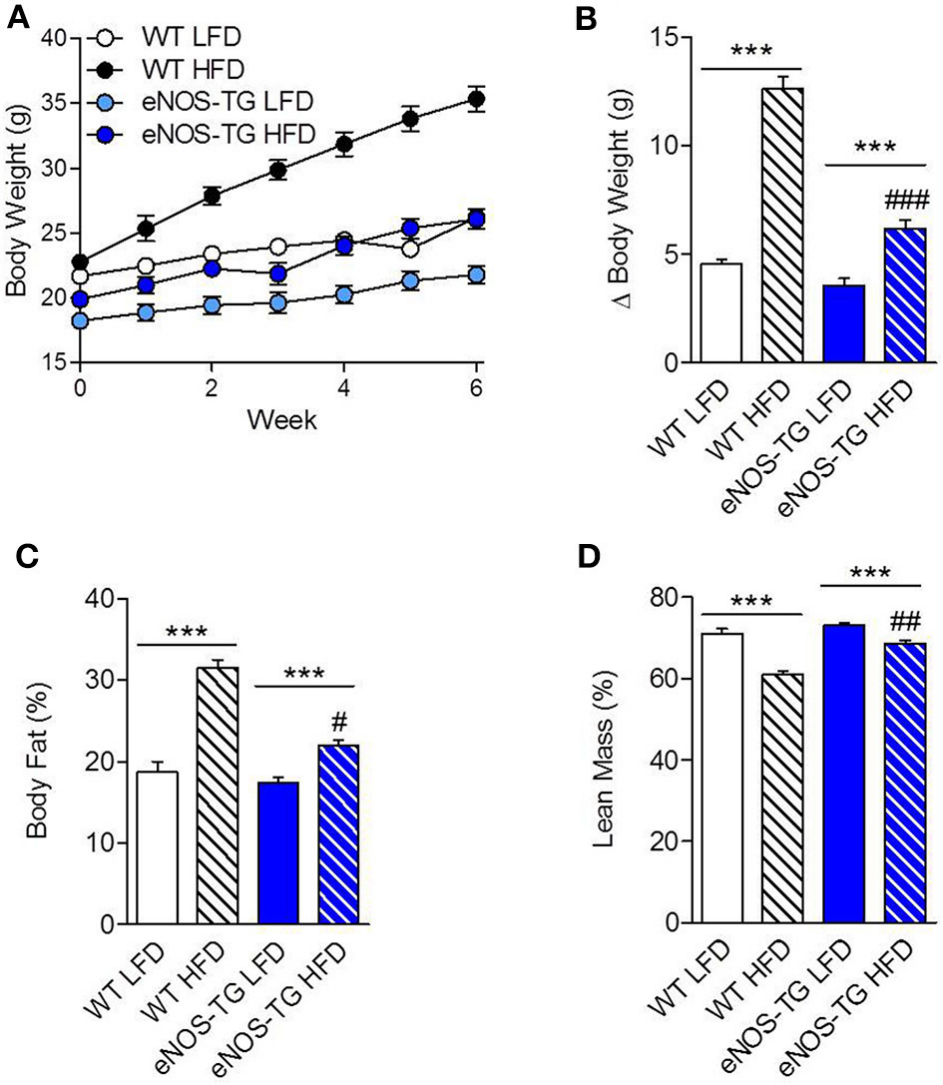
**Overexpression of eNOS prevents diet-induced obesity. (A)** Body weights of wild type (WT) and eNOS-TG mice during 6 weeks of low (LFD) or high fat diet (HFD). **(B)** Summarized weight gain after 6 weeks of LFD or HFD. **(C)** Body fat percentage and **(D)** lean mass percentage following 6 weeks of diet measured by Dexascan analysis. *n* = 7 per group; ^***^p < 0.001 vs. indicated group; ^#^*p* < 0.05, ^##^*p* < 0.01, and ^###^*p* < 0.001 vs. WT HFD.

Wild-type and eNOS-TG mice were placed on a LFD or HFD for 6 weeks. As expected, feeding a HFD led to a two-fold increase in the body weights of WT mice when compared with LFD controls (*p* < 0.001; Figures 1A,B). While there was no difference in weight gain between WT and eNOS-TG mice fed LFD, we found that high fat-fed eNOS-TG mice gained less weight compared with WT mice (Figures 1A,B). Indeed, Dexascan analyses showed significantly less adiposity in high fat-fed eNOS-TG mice than WT mice fed a HFD, with a correspondingly higher percentage of lean mass (Figures 1C,D). This anti-obesogenic phenotype of eNOS-TG mice was shown previously to be independent of changes in food consumption (Sansbury et al., 2012). Collectively, these observations confirm our previous findings that overexpression of eNOS decreases adiposity and prevents weight gain induced by HFD.

### HFD and eNOS overexpression significantly affect the plasma metabolite profile

**Figure 2:**
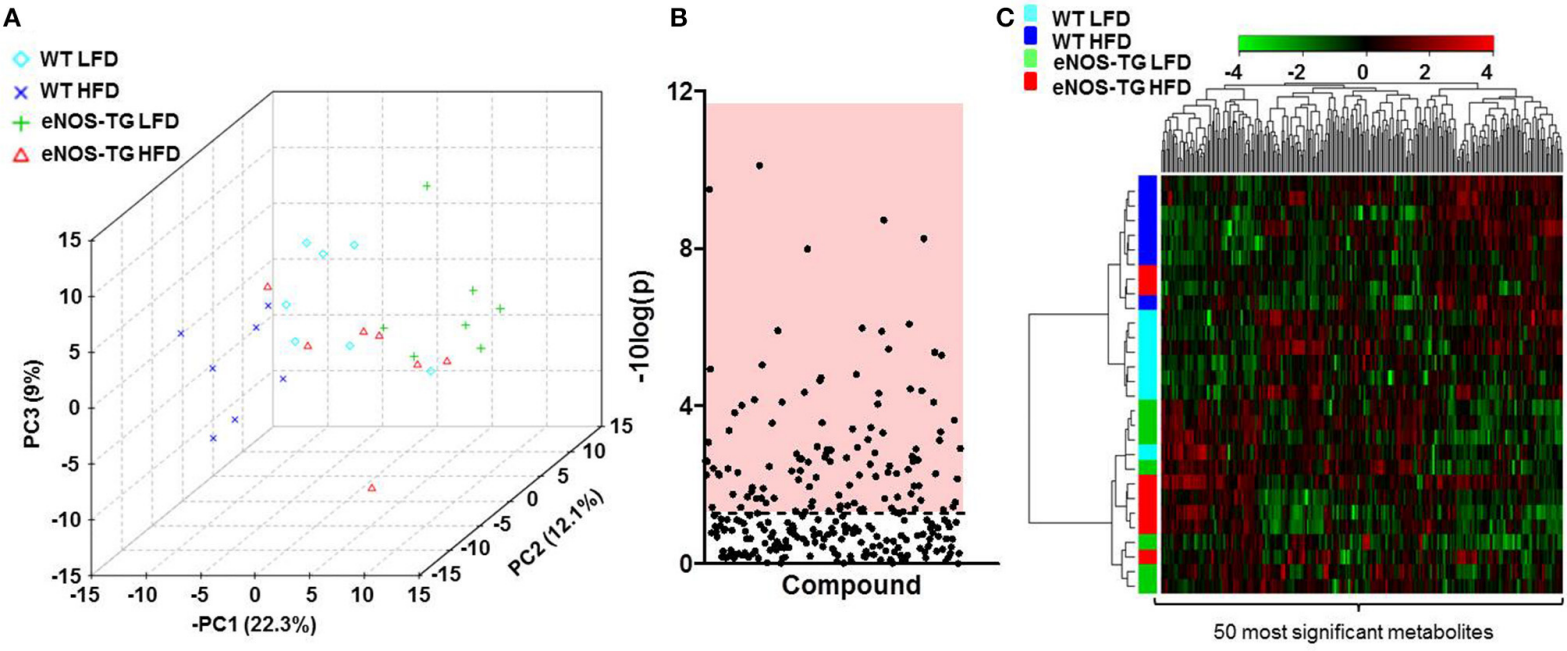
**High fat feeding and eNOS overexpression significantly affect the plasma metabolite profile**. **(A)** Principal component analysis of plasma metabolites of wild type (WT) or eNOS-TG mice fed a low fat (LFD) or high fat diet (HFD). *n* = 7 per group. **(B)** Of the 298 plasma metabolites measured, 116 were determined to be significantly different by ANOVA, and these are displayed in the region of the plot shaded red. Identities of metabolites found to be significantly different are found in Table 1. **(C)** Hierarchical clustering heatmap and dendogram analyses of the 50 most significantly changed plasma metabolites. *n* = 7 per group.

To examine how nutrient excess and eNOS overexpression affect systemic metabolism, we measured the relative levels of 298 different metabolites in the plasma of 7 animals from each of the experimental groups (i.e., WT mice fed a LFD; WT mice fed a HFD; eNOS-TG mice fed a LFD, and eNOS-TG mice fed a HFD) using an unbiased metabolomic approach. Principle component analysis (PCA) was used to identify whether patterns in metabolite abundance were sufficient to separate the groups. As shown in the three-dimensional PCA score plot in Figure 2A, animals within each group tended to cluster together and the metabolite profile was sufficient to discriminate between each experimental group. Of the 298 metabolites measured, ANOVA revealed that 116 metabolites were significantly different (Figure 2B and Table 1), and hierarchical clustering heatmap and dendogram analyses of the 50 most significantly changed metabolites revealed both diet- and genotype-independent clustering (Figure 2C). These analyses suggest that both the composition of the diet and the eNOS transgene differentially regulate the metabolic profile.

**Table 1:** **List of plasma metabolites that changed significantly in WT and eNOS-TG mice fed LFD or HFD**.

**Metabolite**	***p*-Value**	**−10log(p)**	**FDR**
3-dehydrocarnitine	8.03e-11	10.095	2.10e-08
1,5-anhydroglucitol (1,5-AG)	3.14e-10	9.5027	4.10e-08
Mead acid (20:3n9)	1.92e-09	8.7159	1.67e-07
Pyridoxate	5.63e-09	8.2496	3.67e-07
Cinnamoylglycine	1.02e-08	7.9934	5.30e-07
Palmitoleate (16:1n7)	8.34e-07	6.0787	3.63e-05
Hippurate	1.04e-06	5.9835	3.63e-05
Alanylalanine	1.19e-06	5.9227	3.63e-05
Mannose	1.25e-06	5.9024	3.63e-05
Myristate (14:0)	3.54e-06	5.4511	9.24e-05
Stearoyl sphingomyelin	4.28e-06	5.3691	0.000101
Taurodeoxycholate	5.09e-06	5.2931	0.000111
3-indoxyl sulfate	9.15e-06	5.0386	0.000184
17-methylstearate	1.15e-05	4.9406	0.000214
Glycolate (hydroxyacetate)	1.54e-05	4.8131	0.000268
Dihomo-linoleate (20:2n6)	1.89e-05	4.7233	0.000308
Deoxycholate	2.22e-05	4.6541	0.00034
Palmitoyl sphingomyelin	3.66e-05	4.437	0.00053
Propionylcarnitine	4.09e-05	4.3886	0.000561
Cholate	4.43e-05	4.3533	0.000578
Linolenate [alpha or gamma; (18:3n3 or 6)]	4.65e-05	4.3328	0.000578
2-palmitoleoylglycerophosphocholine	6.85e-05	4.1643	0.000813
Stearidonate (18:4n3)	7.85e-05	4.1049	0.000861
Alpha-tocopherol	7.91e-05	4.1016	0.000861
Linoleate (18:2n6)	8.98e-05	4.0466	0.000938
2-eicosatrienoylglycerophosphocholine	9.65e-05	4.0155	0.000969
2-aminoadipate	0.000148	3.8288	0.001434
Urea	0.000229	3.6399	0.002136
Dihomo-linolenate (20:3n3 or n6)	0.000262	3.582	0.00235
7-alpha-hydroxy-3-oxo-4-cholestenoate (7-Hoca)	0.00027	3.5685	0.00235
Isovalerylcarnitine	0.000352	3.4531	0.002966
Glycerol	0.000377	3.4239	0.003073
1-palmitoleoylglycerophosphoethanolamine	0.000416	3.3809	0.00329
Taurocholate	0.000444	3.3526	0.003409
Margarate (17:0)	0.000479	3.3199	0.00357
Isobutyrylcarnitine	0.000715	3.146	0.005181
Taurochenodeoxycholate	0.000747	3.1269	0.005267
13-HODE + 9-HODE	0.000831	3.0805	0.005706
Cysteine	0.001067	2.972	0.007137
Gulono-1,4-lactone	0.001121	2.9505	0.007312
Xylose	0.001214	2.9158	0.007588
Phenol sulfate	0.001221	2.9133	0.007588
Docosapentaenoate (n3 DPA; 22:5n3)	0.0013	2.886	0.007854
Eicosenoate (20:1n9 or 11)	0.001324	2.8781	0.007854
Laurate (12:0)	0.001537	2.8132	0.008917
Butyrylcarnitine	0.001631	2.7875	0.009158
Docosahexaenoate (DHA; 22:6n3)	0.001649	2.7827	0.009158
Pantothenate	0.001918	2.7171	0.010308
Docosadienoate (22:2n6)	0.001935	2.7133	0.010308
Glucose	0.002037	2.6911	0.010632
Caprate (10:0)	0.002129	2.6719	0.010893
Carnitine	0.002204	2.6567	0.011064
Phenylalanine	0.002343	2.6303	0.011536
1-(3-aminopropyl)-2-pyrrolidone	0.002465	2.6082	0.011809
10-nonadecenoate (19:1n9)	0.002507	2.6008	0.011809
Eicosapentaenoate (EPA; 20:5n3)	0.002535	2.596	0.011809
P-cresol sulfate	0.002585	2.5876	0.011809
Cis-vaccenate (18:1n7)	0.002624	2.581	0.011809
Isoleucine	0.003002	2.5227	0.013278
Myristoleate (14:1n5)	0.003304	2.481	0.014339
4-methyl-2-oxopentanoate	0.003351	2.4748	0.014339
1-arachidonoylglycerophosphoinositol	0.003807	2.4194	0.016025
1-palmitoleoylglycerophosphocholine	0.003871	2.4122	0.016036
Palmitate (16:0)	0.004039	2.3937	0.016473
Caprylate (8:0)	0.004348	2.3617	0.017459
Beta-muricholate	0.00446	2.3507	0.017637
Myo-inositol	0.004789	2.3197	0.018657
2-arachidonoylglycerophosphoethanolamine	0.004934	2.3068	0.018937
N-acetylphenylalanine	0.005072	2.2949	0.019184
Stearoylcarnitine	0.005207	2.2834	0.019334
1-eicosatrienoylglycerophosphocholine	0.005259	2.2791	0.019334
10-heptadecenoate (17:1n7)	0.00559	2.2526	0.019851
1-heptadecanoylglycerophosphocholine	0.005621	2.2502	0.019851
Hexadecanedioate	0.005628	2.2496	0.019851
Leucine	0.006128	2.2127	0.021326
Cysteine-glutathione disulfide	0.006518	2.1859	0.022383
Valine	0.007131	2.1469	0.024169
Campesterol	0.007414	2.13	0.024808
Glutamine	0.010255	1.9891	0.033879
Phosphate	0.010449	1.9809	0.034091
Pelargonate (9:0)	0.010797	1.9667	0.034791
Citrate	0.011349	1.9451	0.036104
2-oleoylglycerophosphocholine	0.011481	1.94	0.036104
3-methyl-2-oxobutyrate	0.012311	1.9097	0.038252
2-myristoylglycerophosphocholine	0.014728	1.8319	0.045223
Palmitoylcarnitine	0.016828	1.774	0.051072
1-stearoylglycerophosphocholine	0.017414	1.7591	0.052241
Gamma-glutamylglutamine	0.018021	1.7442	0.053449
2-linoleoylglycerophosphocholine	0.019827	1.7028	0.058143
N-acetyltryptophan	0.020569	1.6868	0.059651
Acetylphosphate	0.021677	1.664	0.062173
3-hydroxyisobutyrate	0.022059	1.6564	0.062581
2-palmitoylglycerophosphoethanolamine	0.022467	1.6484	0.062583
Methionine	0.022539	1.6471	0.062583
Proline	0.024342	1.6136	0.066876
Creatine	0.026059	1.584	0.070849
Uridine	0.027307	1.5637	0.073475
3-ureidopropionate	0.028624	1.5433	0.076232
Glycerophosphorylcholine (GPC)	0.029138	1.5355	0.076819
Gamma-glutamylleucine	0.033609	1.4735	0.087721
Glucuronate	0.033994	1.4686	0.087845
Corticosterone	0.035259	1.4527	0.090223
Urate	0.036642	1.436	0.092769
1-arachidonoylglycerophosphoethanolamine	0.037283	1.4285	0.092769
2-docosahexaenoylglycerophosphoethanol	0.037321	1.428	0.092769
amine			
N1-methyladenosine	0.03905	1.4084	0.096151
Pentobarbital	0.040318	1.3945	0.097653
Histidine	0.040408	1.3935	0.097653
Gamma-glutamylisoleucine	0.041908	1.3777	0.10035
Threonate	0.043121	1.3653	0.10231
Hexanoylcarnitine	0.043571	1.3608	0.10245
EDTA	0.045224	1.3446	0.10539
Bradykinin, des-arg(9)	0.046511	1.3324	0.10743
Oleate (18:1n9)	0.047848	1.3201	0.10948
Cholesterol	0.04824	1.3166	0.10948
N-acetylleucine	0.048898	1.3107	0.11002

*Wild-type (WT) or eNOS-TG mice on C57BL/6J backgrounds were fed a low fat or high fat diet (LFD or HFD, respectively) for 6 weeks. Plasma was then subjected to LC or GC mass spectrometric analysis. Plasma metabolites in mice that were at least 50% changed in abundance and significantly different by One-Way ANOVA are shown below. n = 28, with 7 mice per group*.

### Effects of HFD on the plasma metabolite profile

**Figure 3:**
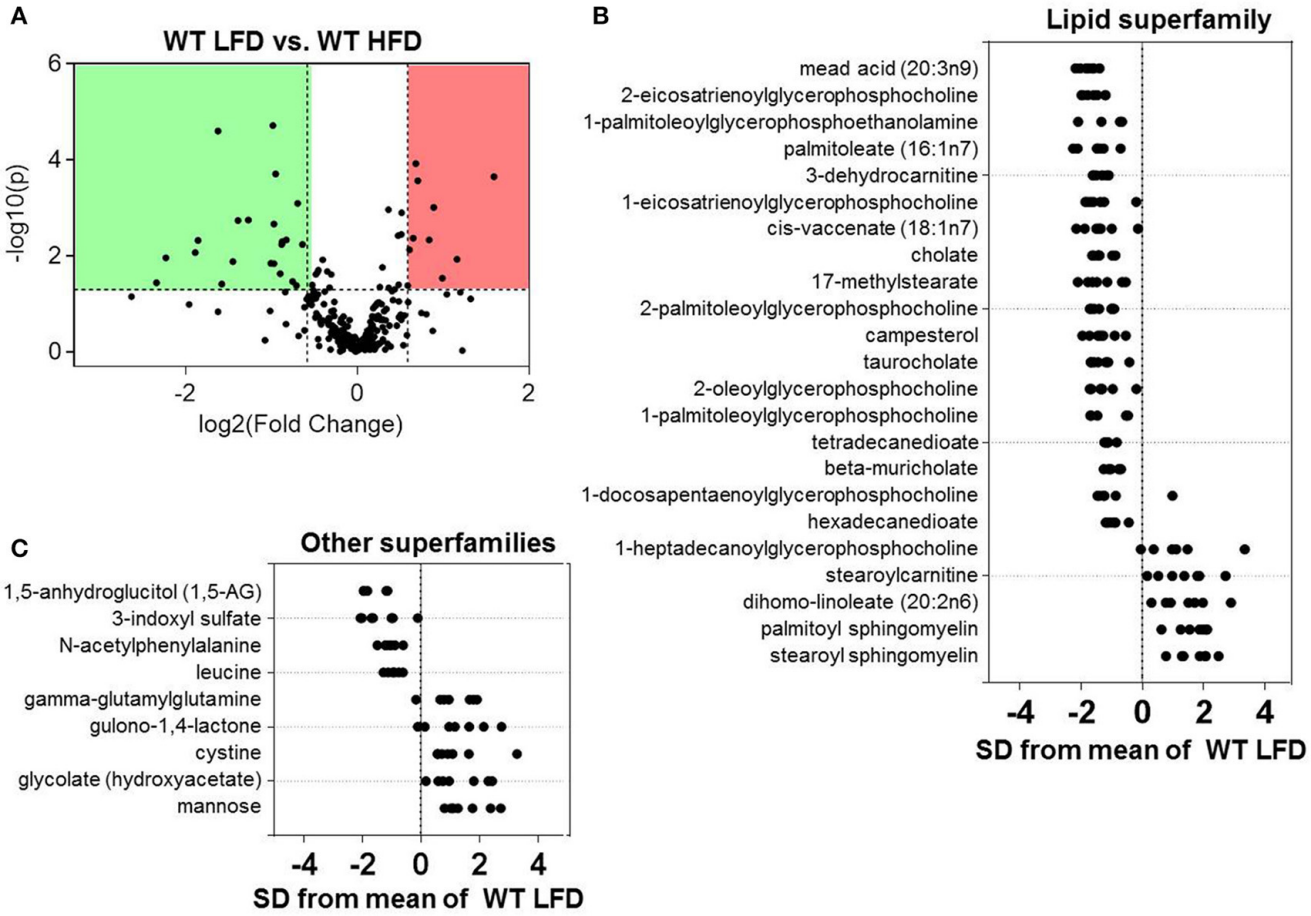
**Effects of high fat feeding on the plasma metabolite profile**. Metabolomic analyses of plasma from wild type (WT) mice fed a low fat (LFD) or high fat diet (HFD) for 6 weeks: **(A)** Volcano plot of metabolites: Those metabolites that increased significantly are in the region of the plot shaded red and those that decreased significantly are in the region shaded green (*p* < 0.05, unpaired *t*-test); **(B,C)** Z-score plot analysis of metabolite changes in plasma from low and high fat-fed mice, separated by metabolite superfamily. Data are shown as standard deviations from the mean of LFD. Only metabolites that increased or decreased significantly and were changed by ≥50% are shown. Each point represents one metabolite in one sample. *n* = 14 animals: 7 WT LFD and 7 WT HFD.

Because we found that both diet and eNOS regulate plasma metabolites, we dichotomized the data and examined the effects of diet in WT mice. Volcano plot analysis showed statistically significant changes in 32 metabolites in mice fed a HFD for 6 weeks (Figure 3A). Further assessment via Z-score plot analysis demonstrated that many of the affected metabolites belonged to the lipid superfamily, of which, the levels of numerous metabolites in the lysolipid (e.g., 2-eicosatrienoylglycerophosphocholine, 1-palmitoleolyglycerophosphoethanolamine, 1-eicosatrienoylglycerophosphocholine, etc.) and bile acid (i.e., cholate, taurocholate, beta-muricholate) subfamilies were lower in the HFD group (Figure 3B). HFD also diminished several long-chain, branched-chain, and dicarboxylate fatty acids. One lysolipid, 1-heptadecanoylglycerophosphocholine, was higher in plasma of high fat-fed mice, as were stearoylcarnitine, dihomolinoleate, and palmitoyl- and stearoyl-sphingomyelin (Figure 3B). Notable decreases in the marker of short-term marker glycemic control, 1-5-anhydroglucitol, were observed in mice fed a HFD (Figure 3C). Also decreased were the amino acid superfamily members: 3-indoxyl sulfate, N-acetylphenylalanine, and leucine. Higher abundance of gamma-glutamylglutamine, gulono-1,4-lactone, cystine, glycolate, and mannose were also observed in the plasma of high fat-fed mice (Figure 3C).

### Effects of eNOS overexpression on the plasma metabolite profile

**Figure 4:**
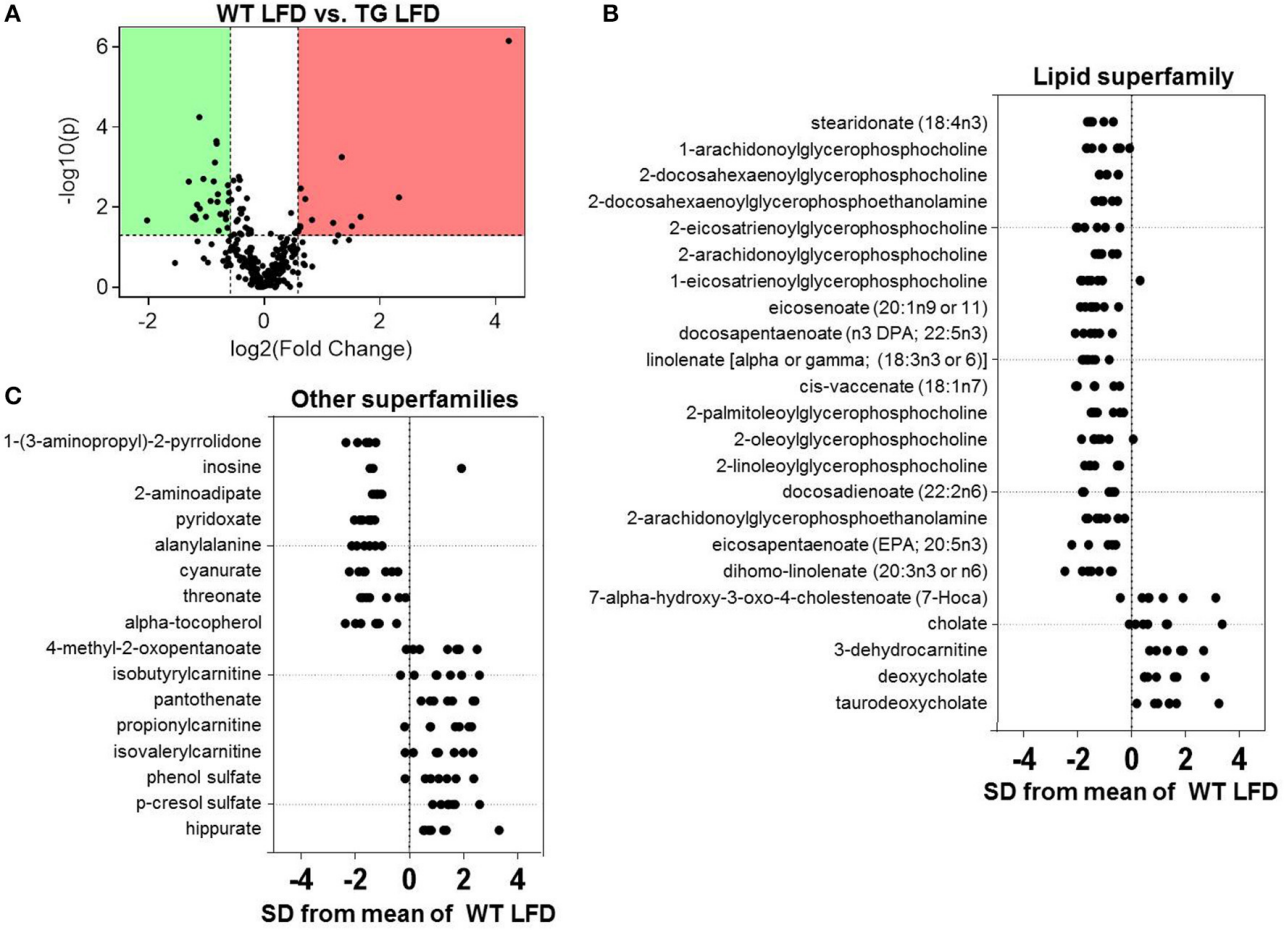
**Effects of eNOS overexpression on the plasma metabolite profile**. Metabolomic analyses of plasma from wild type (WT) and eNOS-TG mice fed a low fat diet (LFD) for 6 weeks. **(A)** Volcano plot of metabolites: Those metabolites that increased significantly are in the region of the plot shaded red and those that decreased significantly are in the region shaded green (*p* < 0.05, unpaired *t*-test); **(B,C)** Z-score plot analysis of metabolite changes in plasma from low fat-fed WT and eNOS-TG mice, separated by metabolite superfamily. Data are shown as standard deviations from the mean of WT LFD. Only metabolites that increased or decreased significantly and were changed by ≥50% are shown. Each point represents one metabolite in one sample. *n* = 14 animals: 7 WT LFD and 7 WT HFD.

To evaluate effects of the eNOS transgene on metabolism, we assessed changes in the plasma metabolome of WT and eNOS-TG mice fed a LFD (Figure 4A). As shown in the Z-score plots in Figure 4B, we found an increase in the plasma levels of 13 metabolites in eNOS-TG mice, including 4 members of the bile acid subfamily (7-α-hydroxy-3-oxo-cholestenoate, cholate, deoxycholate, and taurodeoxycholate). Numerous branched chain amino acid (BCAA) catabolites were higher in abundance in eNOS overexpressing mice as well. These included 4-methyl-2-oxopentanoate as well as several carnitine derivatives of BCAA catabolic products, such as isobutyrylcarnitine, propionlycarnitine, and isovalerylcarnitine. The phenylalanine and tyrosine subfamily members phenol sulfate and p-cresol sulfate were higher in abundance, as was the B vitamin pantothenate (Figure 4C). Lastly, the breakdown product of carnitine, 3-dehydrocarnitine, was markedly increased in eNOS-TG mice compared with WT mice.

Consistent with our previous studies showing a lower abundance of circulating free fatty acids in eNOS-TG mice (Sansbury et al., 2012), several long-chain fatty acids including stearidonate, eicosanoate, docosapentaenoate, linolenate, cis-vaccenate, docosadienoate, eicosapentanoate, and dihomo-linolenate were diminished in low fat-fed eNOS-TG mice compared with WT controls (Figure 4B). Significantly diminished levels of numerous lysolipids, xenobiotics (1-(3-aminopropyl)-2-pyrrolidone, cyanurate), 2-aminoadipate, pyridoxate, alanylalanine, threonate, and α-tocopherol were also observed in plasma from eNOS-TG mice (Figures 4B,C).

### Comparison of plasma metabolites in high fat-fed WT and eNOS-TG mice

**Figure 5:**
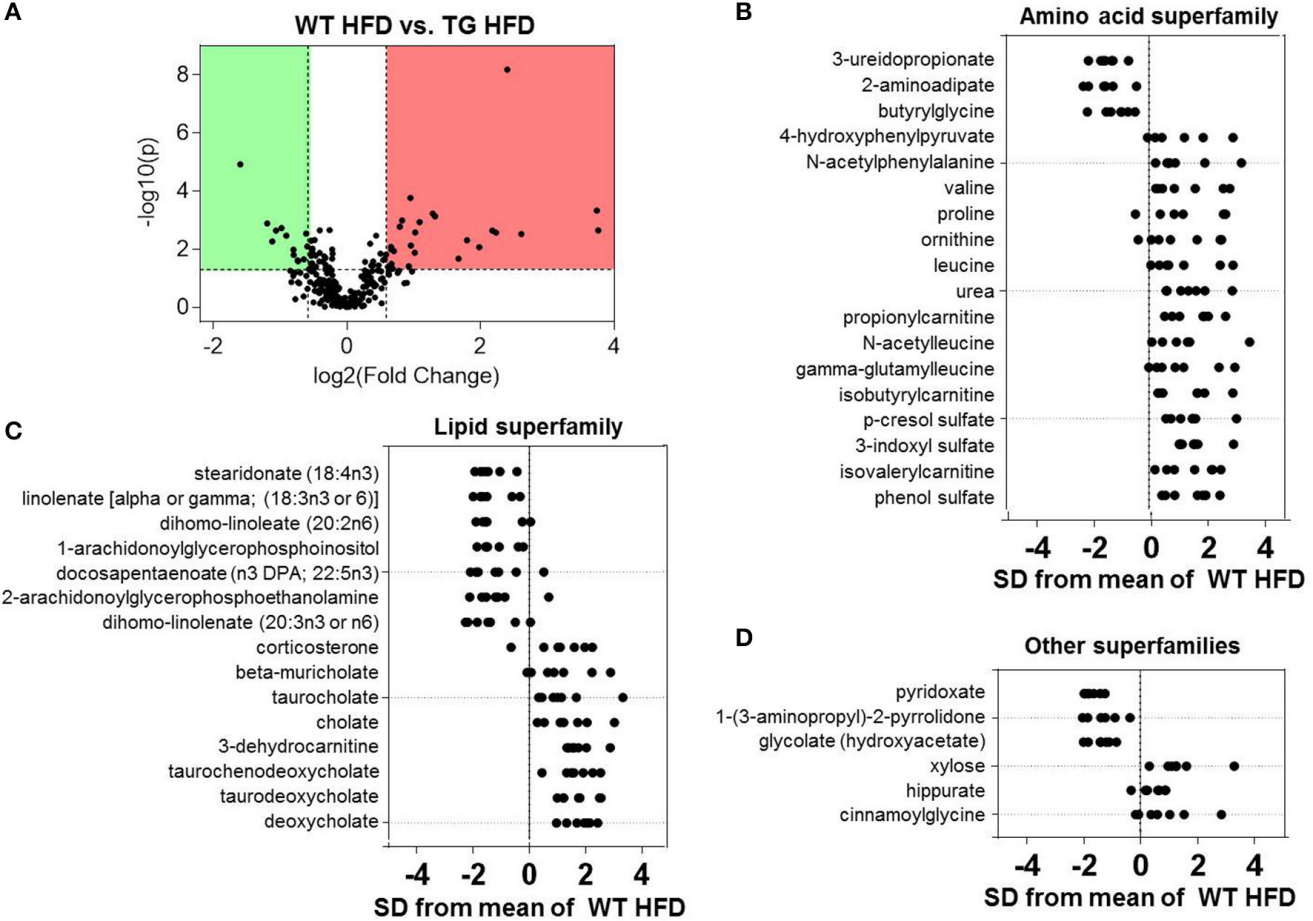
**Comparison of plasma metabolites in high fat-fed wild type and eNOS-TG mice**. Metabolomic analyses of plasma from WT and eNOS-TG mice fed a high fat diet (HFD) for 6 weeks. **(A)** Volcano plot of metabolites: Those metabolites that increased significantly are in the region of the plot shaded red and those that decreased significantly are in the region shaded green (*p* < 0.05, unpaired *t*-test); **(B–D)** Z-score plot analysis of metabolite changes in plasma from high fat-fed WT and eNOS-TG mice, separated by metabolite superfamily. Data are shown as standard deviations from the mean of WT HFD. Only metabolites that increased or decreased significantly and were changed by ≥50% are shown. Each point represents one metabolite in one sample. *n* = 14 animals: 7 WT LFD and 7 WT HFD.

Differences in the plasma metabolite profile between WT and eNOS-TG mice fed a HFD are perhaps most important for understanding the systemic changes in metabolism that could contribute to the anti-obesogenic effects of eNOS. Volcano plot analysis of metabolites from high fat-fed mice showed 39 metabolites that were significantly different between the groups (Figure 5A). Of all superfamilies, the amino acid superfamily was most highly represented, with significant increases in BCAAs (valine, leucine), BCAA catabolites (propionylcarnitine, isobutyrylcarnitine, isovalerylcarnitine), metabolites in the urea cycle (proline, ornithine, urea) and metabolites of the phenylalanine and tyrosine subfamily (4-hydroxyphenylpyruvate, N-acetylphenylalanine, p-cresol sulfate, 3-indoxyl sulfate) (Figure 5B). Z-score analysis revealed that N-acetylleucine, also a metabolic product in the BCAA pathway, and gamma-glutamylleucine, a breakdown product of proteins in general, were also increased in abundance in eNOS-TG mice. Amino acid superfamily members that were decreased in abundance included 3-ureidopropionate, 2-aminoadipate, and butyrylglycine.

**Figure 6:**
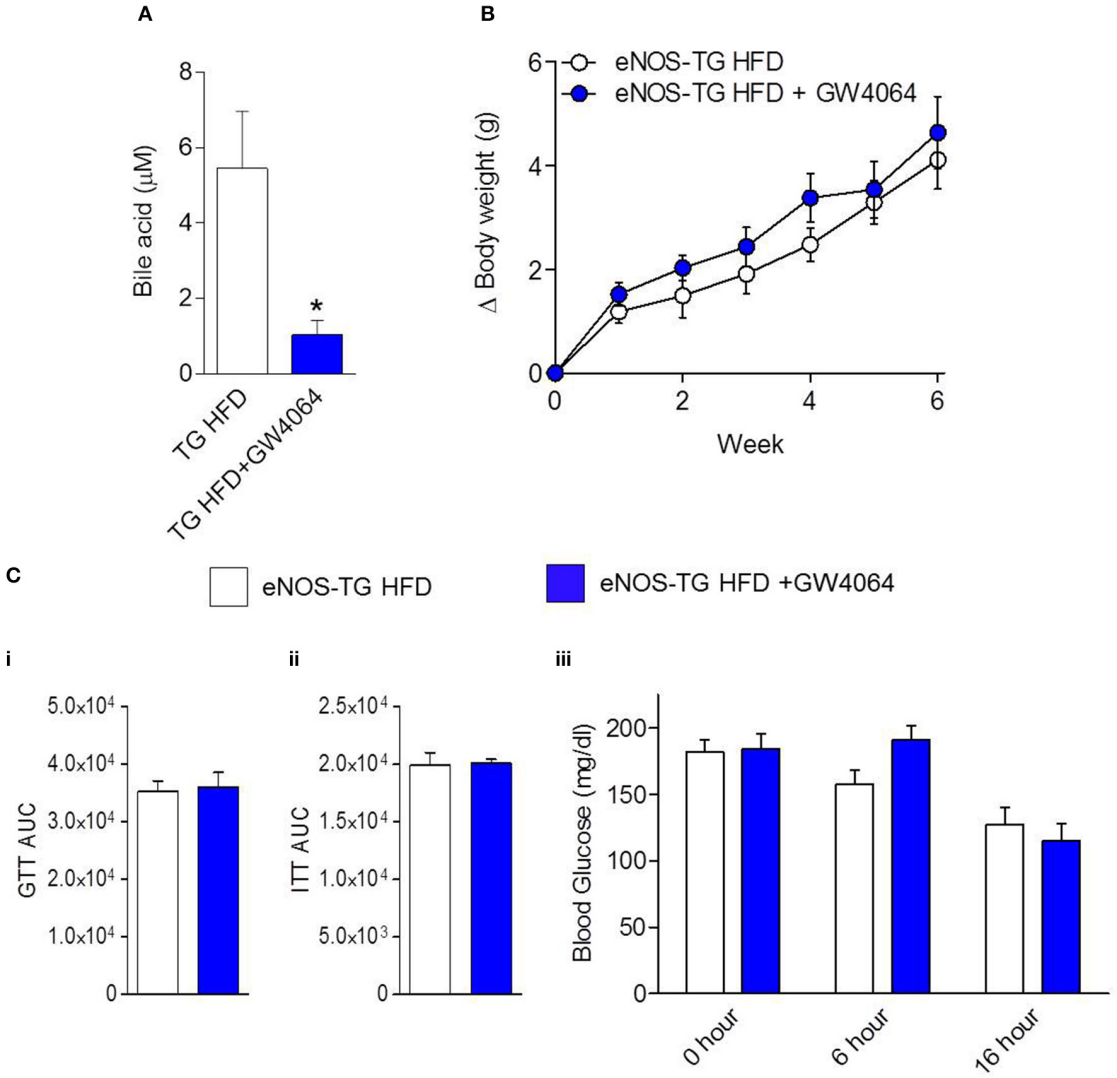
**Inhibition of bile acid synthesis does not promote diet-induced obesity in eNOS-TG mice**. The eNOS-TG mice were fed either a HFD or a HFD containing GW4064 for 6 weeks followed by measurement of: **(A)** Plasma bile acid levels; **(B)** body weight gain; and **(C)** (**i)** fasting blood glucose, **(ii)** glucose tolerance test (GTT) area under the curve (AUC), and **(iii)** insulin tolerance test (ITT) AUC. *n* = 4–6 per group. ^*^p = 0.05 vs. TG HFD.

Plasma from high fat-fed, eNOS overexpressing mice showed lower levels of free fatty acids and some lysolipids compared with WT mice fed a HFD (Figure 5C), similar to changes observed in low fat-fed counterparts (Figure 4). Outside of the lipid and amino acid superfamilies, only 6 metabolites differed in abundance between WT and eNOS-TG mice fed a HFD. Of these, the levels of pyridoxate, 1-(3-aminopropyl)-2-pyrrolidone, and glycolate (Figure 5D) were lower and the levels of xylose, hippurate, and cinnamoylglycine were higher in eNOS-TG than WT mice. The levels of corticosterone and 3-dehydrocarnitine (Figure 5C) were also increased. However, the most striking feature in high fat-fed eNOS-TG mice compared with WT mice fed a HFD was an elevation in nearly all of the bile acids measured (Figure 5C). The levels of beta-muricholate, taurocholate, cholate, taurochenodeoxycholate, taurodeoxycholate, and deoxycholate were increased in eNOS-TG mice fed a HFD. To determine if bile acids are directly responsible for the resistance of eNOS-TG mice to diet-induced weight gain, we administered the farnesoid X receptor (FXR) agonist GW4064, which was shown previously to decrease bile acid abundance and promote obesity in C57BL/6J mice (Watanabe et al., 2011). Despite the fact that plasma bile acid levels were decreased nearly five-fold by GW4064 in eNOS-TG mice (Figure 6A), decreasing bile acid synthesis had no effect HFD-induced weight gain during the 6 week feeding period (Figure 6B) and did not affect fasting blood glucose levels or glucose or insulin tolerance (Figure 6C). These data appear to suggest that bile acids are not directly or exclusively responsible for the resistance to weight gain imparted by high levels of eNOS.

**Figure 7:**
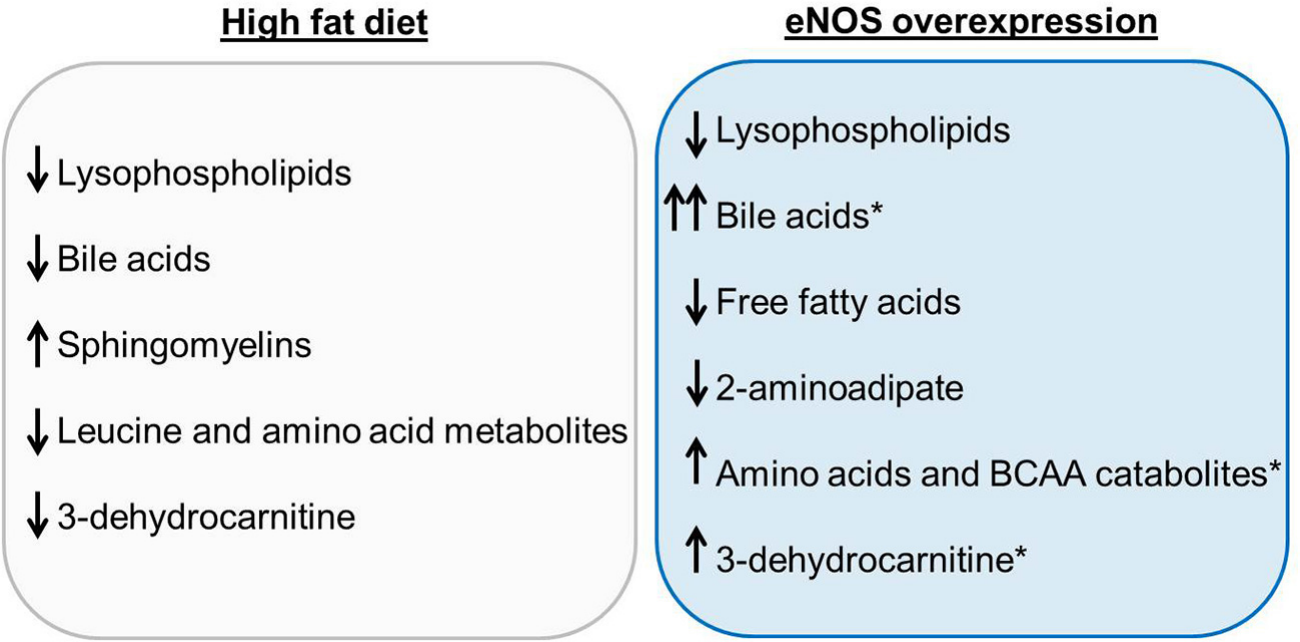
**Summary of the most significant changes in the plasma metabolite profiles due to high fat feeding and eNOS overexpression**. The plasma metabolite changes in wild type (WT) mice fed a high fat diet (HFD) compared with WT LFD are summarized in the gray box. Plasma metabolite changes between WT LFD and eNOS-TG LFD are displayed in the blue box. Metabolites that remained significantly elevated in eNOS-TG HFD compared with WT HFD are indicated by the asterisk.

Collectively, these results help define the impact of nutrient excess and eNOS expression on systemic metabolism (Figure 7). We found that feeding HFD led to significant increases in body weight and adipose tissue mass (Figure 1), changes that were accompanied by an increase in the circulating levels of sphingomyelins and a decrease in the levels of lysolipids, bile acids, some amino acid metabolites, and 3-dehydrocarnitine. Overexpression of eNOS decreased lysolipids, 2-aminoadipate and free fatty acids, but it increased the abundance of bile acids, 3-dehydrocarnitine, as well as BCAAs and their catabolic products. Importantly, the effects of eNOS overexpression on bile acids appeared to be even more prominent when examined in the setting of HFD, although changes in bile acids did not seem to contribute to the lean phenotype of eNOS-TG mice.

## Discussion

The major goals of this study were to assess how nutrient excess alters systemic metabolism and to understand how an increase in NO modifies specific metabolic pathways in the context of diet-induced obesity. Using an unbiased metabolomic approach, we found numerous changes in lipid metabolites caused by a HFD. Most notably, diet-induced obesity in high fat-fed WT mice was accompanied by a decrease in the circulating levels of bile acids and lysolipids. Conversely, eNOS overexpression promoted higher levels of circulating bile acids and also increased the abundance of BCAAs and BCAA catabolic products. Because bile acids have been shown to prevent adiposity, we hypothesized that eNOS overexpression stimulates bile acid synthesis, which in turn provides intrinsic resistance to diet-induced obesity. However, eNOS-TG mice remained lean on a HFD even when bile acid abundance was decreased pharmacologically, suggesting that eNOS-induced increases in bile acids are not solely responsible for the anti-obesogenic phenotype related with high eNOS activity. Nevertheless, these observations do suggest that bile acid production and amino acid metabolism are regulated by NO and that processes that increase bile acids or other metabolic pathways may be important in mediating the anti-obesogenic effects of NO.

The wealth of metabolic changes due to nutrient excess and eNOS provides important information regarding systemic metabolism. In particular, changes due to HFD alone could be important for understanding the metabolic changes that occur due to nutrient excess-mediated obesity. Several metabolic changes indicate an insulin resistant phenotype caused by HFD. For example, HFD promoted lower levels of 1,5-anhydroglucitol (1,5-AG), a marker of glycemic control (Yamanouchi et al., 1996; Stickle and Turk, 1997), which is indicative of systemic insulin resistance and is in accordance with our previous studies showing systemic glucose intolerance and insulin insensitivity in this model (Cummins et al., 2014). As in our previous analysis of adipose tissue (Cummins et al., 2014), we found that the plasma levels of palmitoyl sphingomyelin and stearoyl sphingomyelin were profoundly increased in obese mice. Interestingly, genetic deletion of sphingomyelin synthesizing enzymes appears to protect against diet-induced obesity and insulin resistance (Li et al., 2011; Mitsutake et al., 2011). The breakdown of sphingomyelin could yield significant amounts of ceramide, which is a potent inhibitor of insulin signaling (Chavez and Summers, 2012). Hence, elevated levels of sphingomyelin could be a harbinger of ceramide, which has been shown to be increased by 300% in plasma of high fat-fed mice (Shah et al., 2008) and obese humans (Haus et al., 2009).

Although lysolipids were clearly diminished in plasma from high fat-fed WT mice, the significance of this finding to the phenotypic response to HFD is unclear. Lysophosphatidylcholines (LysoPCs) were most the most common lysolipid affected by HFD in our study. LysoPCs are known to account for 5–20% of all phospholipids in the serum (Kim et al., 2013) and have been suggested to be closely associated with endothelial dysfunction, oxidative stress, inflammation, atherogenesis, and obesity (Kim et al., 2013). In our analysis, several species of lysoPC, including lysoPC 16:1 and 18:1 were decreased while one, lysoPC 17:0, was increased by HFD. These findings are in accordance with a previous study that showed decreased levels of lysoPC 16:1 and 18:1, as well as lysoPC 14:0, 15:0, 16:0, 17:1, 18:2, 19:0, 20:1, and 20:4, while lysoPC 17:0, 18:0, and 18:3 were increased in diet-induced obese mice (Kim et al., 2011). Additional studies have shown decreased serum levels of lysoPC 18:1 and increased lysoPCs 14:0 and 18:0 in obese men (Kim et al., 2010) as well as increased lysoPC 18:0 in high fat-fed pigs (Galili et al., 2007). It is possible that the abundance of dietary lipids contained in the HFD caused an increase in some forms of lysolipids, such as lysoPC 17:0, which consists of one chain of margaric acid (derived from lard) at the C-1 position. This could support the idea that phospholipid remodeling took place in mice consuming HFD. However, several of the same phospholipids were decreased in low fat-fed eNOS-TG mice (Figure 4), which argues against the idea that such lysolipid changes contribute to diet-induced obesity.

While no significant changes in fatty acids were observed in high fat-fed WT mice, stearoylcarnitine was increased, which could suggest an impairment of fat oxidation capacity. In eNOS overexpressing mice, which exhibit increased energy expenditure (Sansbury et al., 2012), decreases in numerous long-chain fatty acids were observed. This is likely due, at least in part, to increased utilization of fats, as genetic deletion of eNOS is known to impair fat oxidation capacity (Le Gouill et al., 2007). It is also possible that eNOS overexpression could inhibit fatty acid synthesis in the liver (Sansbury and Hill, 2014). Nevertheless, the root cause for such eNOS-induced changes in systemic fat metabolism is still unclear.

Because of the established links of bile acid signaling on energy expenditure (Watanabe et al., 2006), lipid and glucose homeostasis (Watanabe et al., 2004; Zhang et al., 2006), and obesity (Watanabe et al., 2006, 2011), we reasoned that changes in the bile acid pathway could be important for the hypermetabolic and anti-obesogenic effects of eNOS. Our analyses show that HFD decreases bile acid abundance in WT mice and that overexpression of eNOS is sufficient to augment bile acid levels. The finding that eNOS increases plasma levels of bile acids is supported by studies showing that perfusion of livers with NO donors increases bile acid outflow acutely (Trauner et al., 1997, 1998), while inhibition of NOS reduces the biosynthesis of bile acids by inhibiting the activity of hepatic Cyp7A1 (Khedara et al., 1998). Nitric oxide has also been shown to inhibit bile acid uptake in hepatocytes (Schonhoff et al., 2011). Metabolomic analyses showed that both primary and secondary bile acids, e.g., cholate and deoxycholate, as well as conjugated bile acids, e.g., taurodeoxycholate, taurocholate, taurochenodeoxycholate, were increased in eNOS-TG mice, suggesting that their hepatic synthesis, bacterial dehydroxylation occurring in the gut, and conjugation in the liver are increased under conditions of higher eNOS activity.

Notably, bile acids have been shown to lower triglycerides by regulating FXR (Watanabe et al., 2004), and decreasing bile acid pool size worsens obesity and diabetes in high fat-fed mice (Watanabe et al., 2011). Moreover, mice overexpressing Cyp7A1, a rate-limiting enzyme in bile acid synthesis (Lefebvre et al., 2009), are resistant to diet-induced obesity, show elevated systemic metabolism, and express higher levels of fat oxidation genes (Li et al., 2010). In clinical trials, dyslipidemic patients given bile acid-sequestering resins exhibited increased plasma triglyceride and VLDL levels (Angelin et al., 1987; Crouse, 1987), and patients with deficiencies in *CYP7A1* are characterized by increased plasma triglyceride concentrations (Pullinger et al., 2002). Hence, we considered that eNOS-mediated resistance to obesity and enhanced fat metabolism might be due to NO-regulated elevations in the abundance of bile acids. However, providing the synthetic FXR agonist GW4064—shown previously to be sufficient to decrease bile acid levels and energy expenditure thereby accentuating diet-induced weight gain and insulin resistance in mice (Watanabe et al., 2011)—was sufficient to decrease bile acids in eNOS-TG mice; however, the GW4064-supplemented eNOS-TG mice maintained their lean phenotype on HFD, and the drug did not affect glucose tolerance or insulin sensitivity.

While these data suggest a bile acid-independent mechanism for the anti-obesogenic effect of eNOS, there are several possibilities and limitations to consider. For example, it is possible that while bile acids are not directly responsible for promoting leanness in eNOS-TG mice, effects secondary to their elevation could play a role. For example, recent studies have unveiled important roles for bile acids in regulating gut microbiota, which are proving important for regulating energy metabolism, adiposity, and insulin resistance (Li and Chiang, 2014). Hence, not only are intestinal bacteria important for the synthesis of secondary bile acids, but bile acids themselves could influence the microbiome and impact systemic metabolism. Although previous studies show that dietary administration of GW4064 for as little as 3 weeks significantly increased diet-induced weight gain (Watanabe et al., 2011), it is possible that the 6 week administration used in this study, although sufficient to decrease bile acid abundance, was insufficient to reprogram metabolic changes caused by bile acids, such as differential expression of intestinal microbiota. Moreover, bile acids play an important role in xenobiotic metabolism (Chiang, 2013; Li and Chiang, 2014), which can orchestrate energy metabolism (Gao and Xie, 2012) and could alter obesogenic programs, even prenatally (Janesick et al., 2014). Interestingly, eNOS overexpression was associated with altered levels of several xenobiotic metabolites, such as hippurate, 1-(3-aminopropyl)-2-pyrrolidone, glycolate, cyanurate, and cinnamoylglycine. Hence, while these studies appear to rule out a direct causal effect of bile acids on eNOS-TG-induced resistance to obesity, further studies are required to dissect their secondary or long-term metabolic effects.

Other metabolic pathways affected by overexpression of eNOS may also be important for regulating adiposity. In our analysis, plasma levels of BCAAs and/or their short-chain catabolites (e.g., 4-methyl-2-oxopentanoate) and acyl carnitine derivatives (e.g., isobutyrylcarnitine, propionylcarnitine, isovalerylcarnitine) were decreased in high fat-fed WT mice and increased in eNOS-TG mice. This may be important because BCAA supplementation has been shown to have favorable effects on diet-induced metabolic disease. For example, leucine supplementation prevents obesity in rodents (Zhang et al., 2007; Vianna et al., 2012) and is associated with lower adiposity in humans (Qin et al., 2011), while isoleucine was shown to decrease tissue triglyceride accumulation and adiposity and increase expression of PPARα and uncoupling proteins in mice (Nishimura et al., 2010). Additionally, increasing BCAA levels by deletion of BCATm, the enzyme that catalyzes the first step in BCAA catabolism, completely prevents HFD-induced insulin resistance and adiposity in mice, which was suggested to be due to a futile protein turnover cycle (She et al., 2007). That this form of futile cycling might occur in eNOS-overexpressing mice is suggested by elevations of not only BCAAs, but by increased abundance of protein breakdown products such as gamma-glutamylleucine as well. Furthermore, short-chain acylcarnitines, such as propionylcarnitine, were elevated in eNOS-TG mice, and these have been shown to promote mitochondrial biogenesis and increase energy expenditure (Siliprandi et al., 1965; Sayed-Ahmed et al., 2000; She et al., 2007; D'Antona et al., 2010). It should be noted, however, that BCAAs have also been associated with increased insulin resistance and metabolic risk factors in humans as well as rodents (Newgard et al., 2009; Adams, 2011; Cheng et al., 2012). Thus, the role of eNOS-induced changes in BCAAs and BCAA metabolites on adiposity and insulin resistance requires further clarification. Beyond BCAAs, eNOS-TG mice displayed several other metabolic changes that could possibly play a role in regulating obesity. For example, the eNOS-regulated increases in corticosterone or 3-dehydrocarnitine may be indicative of processes related with steroidal- or carnitine-mediated processes that promote energy expenditure and prevent excessive weight gain. Of interest, 2-aminoadipate was decreased by eNOS and this metabolite has been shown to be a biomarker for diabetes risk and to regulate insulin and glucose homeostasis (Wang et al., 2013; Wu et al., 2014). The importance of each of these pathways in mediating the anti-obesogenic effects of eNOS remains to be tested.

In summary, this study identified significant changes due to both nutrient excess and eNOS expression. High fat feeding in WT mice diminished the abundance of lysophospholipids, bile acids, BCAAs and their metabolites, and 3-dehydrocarnitine; and HFD increased metabolic markers of insulin resistance and oxidative stress. Overexpression of eNOS resulted in low levels of numerous free fatty acids even in the face of nutrient excess and led to remarkable elevations in the levels of bile acids and BCAAs. These results further delineate systemic metabolic changes induced by high fat diet and eNOS overexpression, which could be built upon to develop targeted interventions to thwart the obesity epidemic.

### Conflict of interest statement

The authors declare that the research was conducted in the absence of any commercial or financial relationships that could be construed as a potential conflict of interest.
